# Pathological cardiac remodeling occurs early in CKD mice from unilateral urinary obstruction, and is attenuated by Enalapril

**DOI:** 10.1038/s41598-018-34216-x

**Published:** 2018-10-31

**Authors:** Onju Ham, William Jin, Lei Lei, Hui Hui Huang, Kenji Tsuji, Ming Huang, Jason Roh, Anthony Rosenzweig, Hua A. Jenny Lu

**Affiliations:** 1 Center for Systems Biology, Program in Membrane Biology, Division of Nephrology, Massachusetts General Hospital and Harvard Medical School, Boston, MA 02114 USA; 2 Department of Pharmacology, School of Basic Medical Sciences, Peking University, Beijing, China; 3 College of Arts & Sciences, Washington University in St. Louis, St. Louis, MO 63130 USA; 4 Corrigan Minehan Heart Center, Massachusetts General Hospital and Harvard Medical School, Boston, MA 02114 USA

## Abstract

Cardiovascular disease constitutes the leading cause of mortality in patients with chronic kidney disease (CKD) and end-stage renal disease. Despite increasing recognition of a close interplay between kidney dysfunction and cardiovascular disease, termed cardiorenal syndrome (CRS), the underlying mechanisms of CRS remain poorly understood. Here we report the development of pathological cardiac hypertrophy and fibrosis in early stage non-uremic CKD. Moderate kidney failure was induced three weeks after unilateral urinary obstruction (UUO) in mice. We observed pathological cardiac hypertrophy and increased fibrosis in UUO-induced CKD (UUO/CKD) animals. Further analysis indicated that this cardiac fibrosis was associated with increased expression of transforming growth factor β (TGF-β) along with significant upregulation of Smad 2/3 signaling in the heart. Moreover early treatment of UUO/CKD animals with an angiotensin-converting-enzyme inhibitor (ACE I), Enalapril, significantly attenuated cardiac fibrosis. Enalapril antagonized activation of the TGF-β signaling pathway in the UUO/CKD heart. In summary our study demonstrates the presence of pathological cardiac hypertrophy and fibrosis in mice early in UUO-induced CKD, in association with early activation of the TGF-β/Smad signaling pathway. We also demonstrate the beneficial effect of ACE I in alleviating this early fibrogenic process in the heart in UUO/CKD animals.

## Introduction

Chronic kidney disease (CKD) is a major health problem worldwide. According to the United States Renal Data Service’s 2015 annual report, the overall prevalence of CKD in the general population is approximately 14% (www.niddk.nih.gov/kidney-disease). Both end stage renal disease (ESRD) that requires dialysis and CKD carry high mortality and morbidity, which is largely driven by concomitant cardiovascular disease (CVD). The prevalence of CVD in CKD patients is nearly 70%, which is almost double the prevalence of CVD among non-CKD populations. Additionally more than half of the mortality associated with kidney diseases results from CVD^1–3^. Heart failure and ischemic heart disease (IHD) are the most common causes of CVD-related death in CKD patients^4^, which often lead to a process known as cardiorenal syndrome (CRS). CRS is the coexistence of acute and/or chronic kidney disease and cardiac dysfunction, with failure of one organ accelerating the progression of structural damage and failure in the other organ^5^. CRS has been classified into five categories by Ronco *et. al*. based on event order and time frame of organ failure, after a conference consensus of the Acute Dialysis Quality Initiative^6^. According to this classification acute and chronic kidney insults are termed CRS type 3 and type 4, respectively^6^.

The high prevalence of cardiovascular complications in CKD patients is believed to result from the cumulative effects of hemodynamic overload, anemia, metabolic abnormalities, neuroendocrine deregulation, and inflammatory activation, which are associated with uremia in CKD and ESRD patients^7–11^. Cardiac hypertrophy and fibrosis are frequently observed in the cardiomyopathy associated with CKD and ESRD. This pathological cardiac hypertrophy and fibrosis commonly involves the activation and proliferation of cardiac fibroblasts and the expansion of extracellular matrix (ECM) including collagen I, collagen III, and fibronectin, leading to distorted organ architecture and contractile dysfunction^12–17^. Interestingly, diffuse myocardial fibrosis is considered to be a key feature of the cardiomyopathy seen in uremic patients^18^. There are multiple signaling cascades that mediate cardiac fibrosis including transforming growth factor β (TGF-β), endothelin, and the RhoA–MRTF–SRF signaling pathway. Among them, the TGF-β family remains the master regulator of fibroblast activity and fibrogenesis as is the case in other organ systems^13,19–22^. The initial insults that lead to pathological cardiac remodeling and fibrosis reportedly involve cardiomyocyte senescence, activation of inflammatory cells and cytokines, and overstimulation of the neuroendocrine system especially the renin–angiotensin system (RAS)^23–26^.

The underlying pathophysiology of CRS is extremely complex and poorly understood^27^. Most published studies have utilized various animal models to delineate the underlying mechanism of various subtypes of CRS^28–30^. Most CKD models that are applied in the CRS type 3 and 4 studies use nephrectomy or bilateral ischemia-reperfusion injury to create significant kidney dysfunction and uremia^31–34^. UUO-induced CKD has not been reported in animal studies of type 3 and/or type 4 CRS. It is not known whether pathological cardiac remodeling also occurs in the presence of early mild-to-moderate renal dysfunction from UUO. Acute and chronic urinary obstruction from benign prostate hypertrophy, kidney stones, or various other urinary retentions are commonly seen in the clinical setting. The potential impact of UUO-associated CKD on cardiac remodeling/fibrosis has not been studied in patients or animals. Interestingly, a recent cross-sectional study has uncovered that even without overt cardiac dysfunction, there is still impairment in peak cardiac performance and cardiac functional reserve in asymptomatic CKD patients in the absence of other comorbidities. This indicates that an insidious pre-clinical cardiac pathophysiological process may occur early on in the development of CKD^35^. Therefore, to examine a possible pre-clinical cardiomyopathy in early CKD and understand its underlying molecular mechanisms, we developed an early non-uremic CKD mouse model made through unilateral urinary obstruction, and analyzed the cardiac structure and function. Our study demonstrated that an early maladaptive cardiac hypertrophy with fibrosis occurred in an early stage of non-uremic CKD, which was induced by UUO. Specifically, despite the absence of overt cardiac dysfunction significant cardiac hypertrophy and myocardial fibrosis were observed, in association with an up-regulation of the canonical TGF-β signaling cascade in the hearts of the UUO-induced CKD (UUO/CKD) mice. Moreover, blockade of the RAS with an angiotensin-converting-enzyme inhibitor (ACE I), Enalapril, significantly attenuated the UUO-induced cardiac hypertrophy and fibrosis and down-regulated TGF-β signaling.

## Results

### Chronic kidney injury and systemic hypertension are induced by unilateral urinary obstruction (UUO)

**Figure 1:**
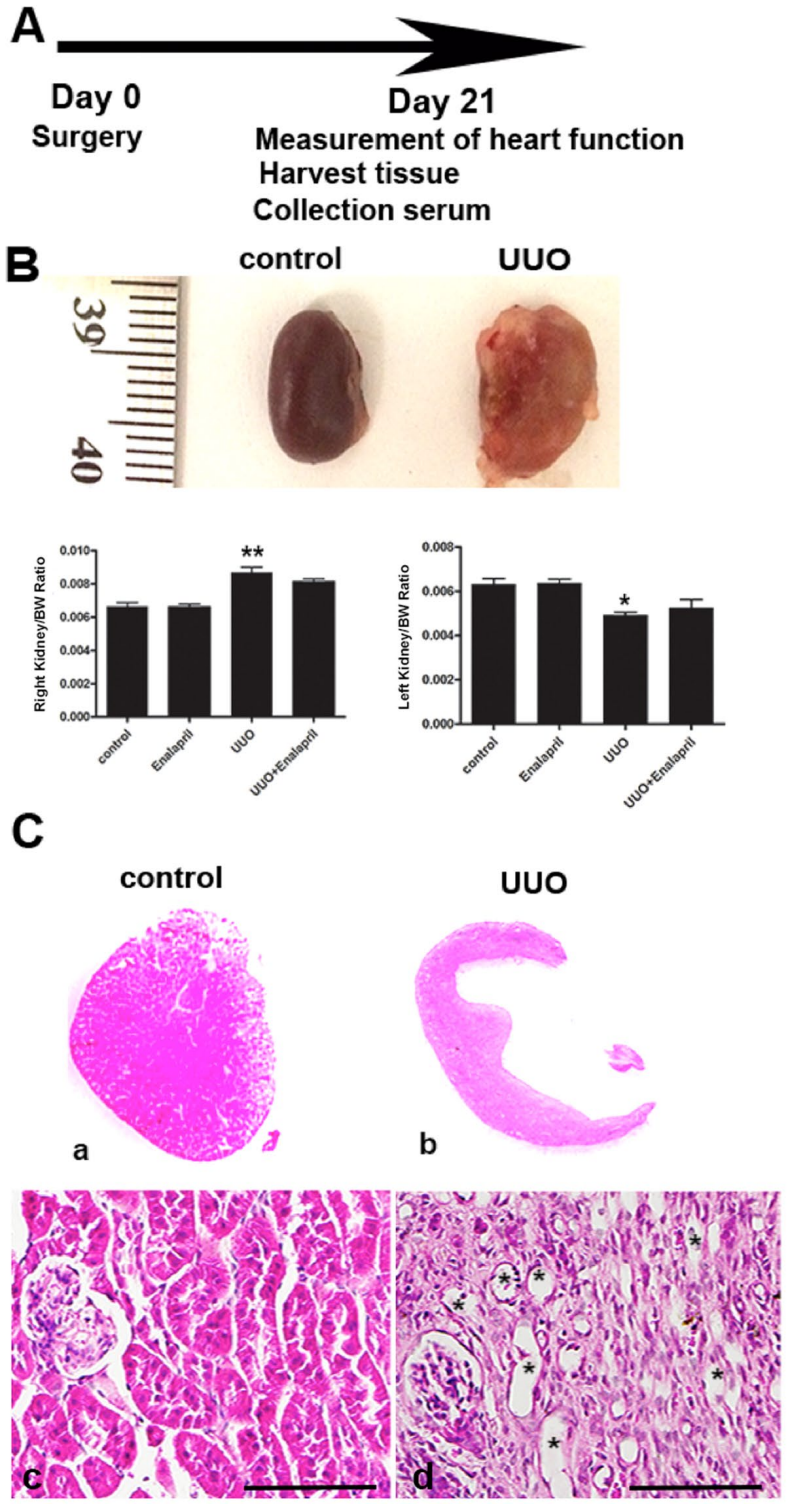
Obstructive nephropathy is induced by unilateral urinary obstruction (UUO) injury in mice. (**A**) Diagram of the time-course of induction of CKD following UUO injury. (**B**) Representative images of non-obstructed right kidney (left panel) and obstructed left kidney (right panel) in mice 3 weeks after UUO injury. Weights of right and left kidneys were measured in the control, Enalapril, UUO, and UUO + Enalapril groups. Graph represents the ratios of the right and left kidney to body weight, respectively. *P < 0.05, **P < 0.01 vs. Control. Statistical analyses were performed with one-way ANOVA. N = 12. (**C**) H&E staining revealed hydronephrosis of the left kidney following UUO injury. Kidney medullary and papillary regions were distorted, with significant atrophy in the UUO mice kidneys compared to the controls. H&E staining of regions containing residual renal cortex showed broadly dilated kidney tubules in the UUO kidneys (indicated by * in (**C**). Magnification: 2 × (upper panels, A and B) and 20× (lower panels, C and D). Bar = 100 μm. Bar values represent means ± SEM (error bars). Statistical analyses were performed with t-tests. N = 8.

UUO is known to cause chronic kidney injury in mice^36^. We performed left UUO in 10–12 week old male (C57BL/6) mice. By three weeks post-ligation of the left ureter significant hydronephrosis was observed in the obstructed left kidney in mice (Fig. 1A). The weight of the non-obstructed right kidney was significantly increased in the UUO mice compared with that of the controls. We further checked whether an ACE I, Enalapril, has therapeutic effects in UUO/CKD mice. After treatment with Enalapril in the UUO/CKD mice the weight of the right kidney was slightly decreased compared to the UUO-only mice, but there was no statistically significant difference. The weight of the obstructed left kidney was decreased indicating loss of renal mass in the obstructed kidney (Fig. 1B). H&E staining revealed the presence of dilated cortical kidney tubules and medullar atrophy in the obstructed left kidney, which is consistent with obstructive nephropathy (Fig. 1C).

**Figure 2:**
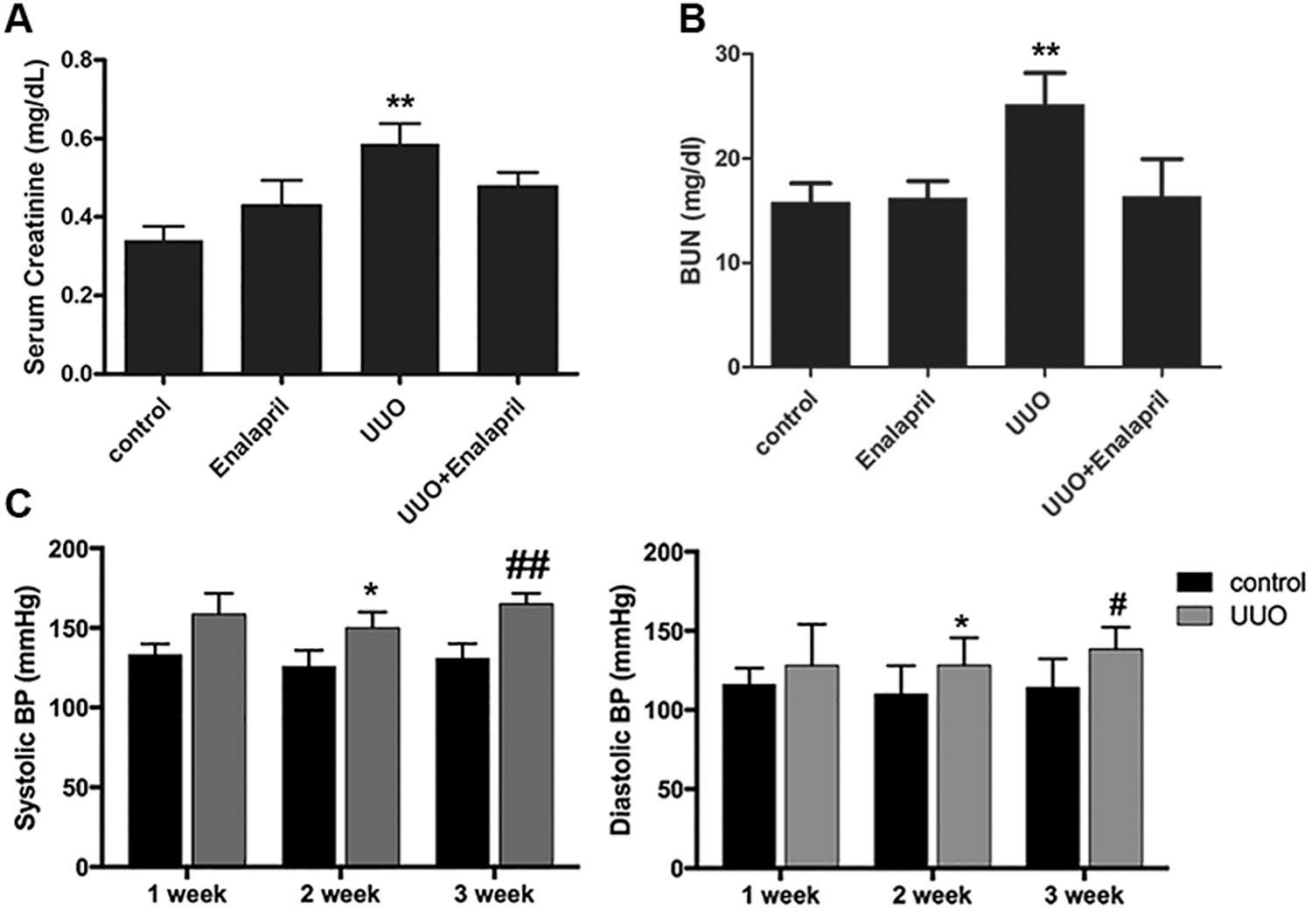
UUO injury causes moderate kidney dysfunction and elevated blood pressure in mice. (**A**) Serum creatinine for the UUO mice was significantly elevated 3 weeks after UUO injury, compared to the controls. Serum creatinine was 0.56 ± 0.02 mg/dL in the UUO-only mice and 0.48 ± 0.36 mg/dL in Enalapril-treated UUO mice, versus 0.3 ± 0.04 mg/dL in the controls. Enalapril treatment alone did not significantly increase serum creatinine in the wild type. Enalapril-treated UUO mice had improved serum creatinine. **P < 0.01 vs. Control. (**B**) Blood urea nitrogen (BUN) was measured. BUN was significantly elevated in UUO mice 3 weeks after injury, compared to the controls. Enalapril treatment prevented elevation of BUN. Enalapril alone did not significantly increase BUN compared to the controls. **P < 0.05 vs. Control. (**C**) Both systolic and diastolic blood pressures (BP) were significantly elevated in the UUO mice starting 2 weeks after UUO injury. The mean systolic BP was 142.33 mmHg and 144.92 mmHg in the UUO mice 2 and 3 weeks after UUO injury respectively, compared to a mean systolic BP of 124.01 mmHg in controls. *P < 0.05 vs. Control for 2-week systolic BP, ^##^*P* < 0.01 vs. Control for 3-week systolic BP, *P < 0.005 vs. Control for 2-week diastolic BP, ^#^*P* < 0.005 vs. Control for 3-week diastolic BP. Bar values represent means ± SEM (error bars). Statistical analyses were performed with t-tests. N = 12.

Serum creatinine was elevated in the UUO mice, to 0.56 ± 0.02 mg/dL compared with 0.35 ± 0.04 mg/dL in the controls, indicating moderate kidney dysfunction in the UUO mice. After Enalapril treatment the serum creatinine improved to 0.48 ± 0.36 mg/dL in the Enalapril-treated UUO/CKD mice (Fig. 2A). Blood urea nitrogen (BUN) levels were also measured. BUN was significantly increased to 25.17 ± 3.03 mg/dL in the UUO/CKD mice, compared with 15.77 ± 1.84 mg/dL in the controls. Enalapril treatment in UUO/CKD mice prevented the elevation of BUN. Enalapril alone did not significantly increase BUN compared to the controls (Fig. 2B). Both systolic and diastolic blood pressures (BP) were significantly elevated in the UUO mice. The systolic blood pressure ranged from 126 mmHg to 185 mmHg with an average of 144.92 mmHg. The diastolic blood pressure ranged from 126 mmHg to 150 mmHg with an average of 124 mmHg. There was an average systolic BP elevation of 20.91 mmHg and diastolic BP elevation of 29.54 mmHg in the UUO mice with moderate CKD compared to the controls (Fig. 2C).

### UUO induces pathological cardiac hypertrophy in mice

**Figure 3:**
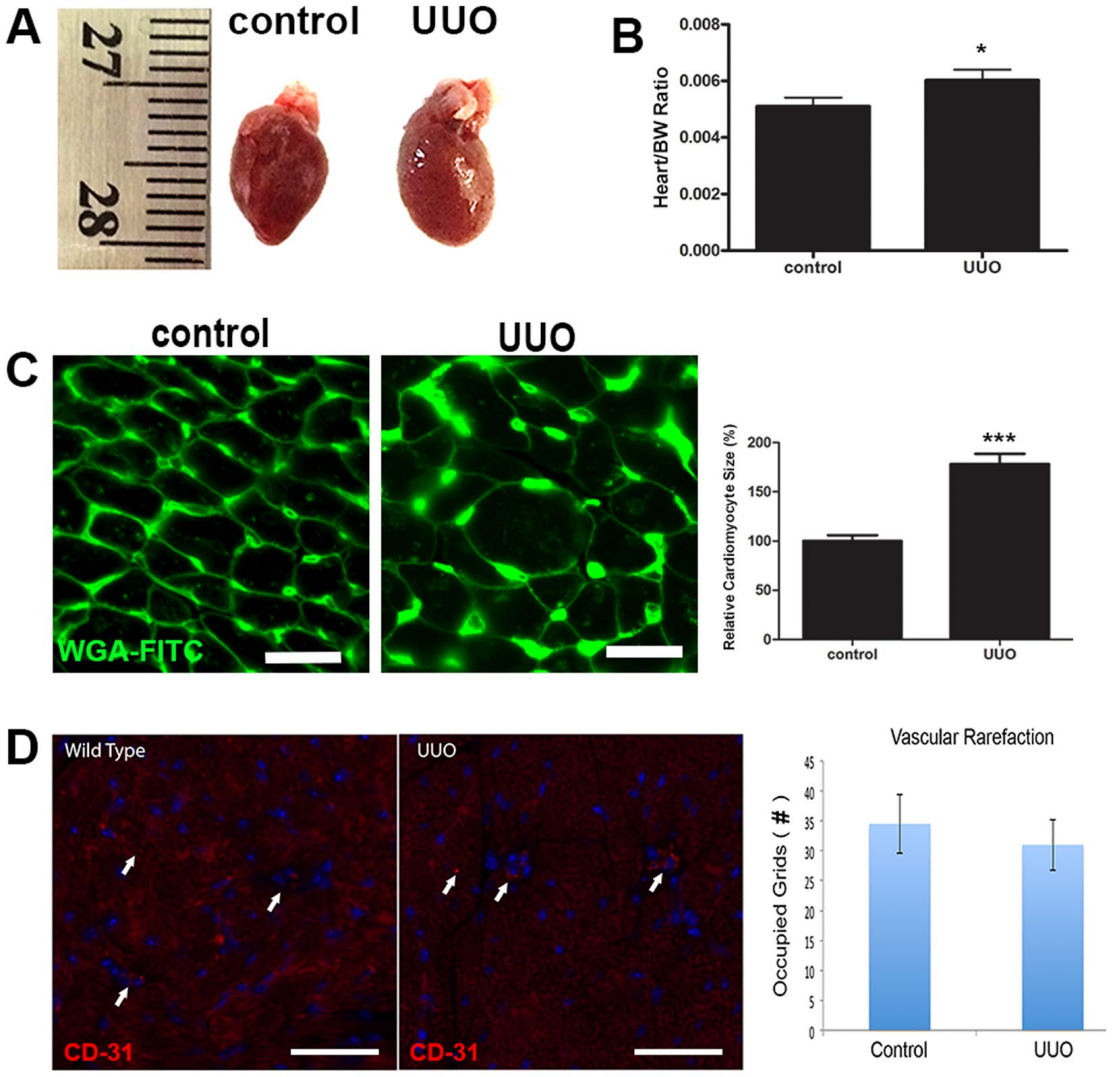
Cardiac hypertrophy developed in UUO mice 3 weeks after injury. (**A**) Representative images of hearts harvested from control mice and mice that underwent UUO. By measurement the heart is slightly enlarged in mice with UUO. (**B**) Cardiac mass as measured by the ratio of heart weight to body weight was significantly increased in the UUO mice. *P < 0.05 vs. Control. N = 12. (**C**) Immunofluorescence staining of cardiac tissues with WGA-conjugated FITC in the control and UUO mice. Significantly increased cardiomyocyte circumference was observed in the UUO mice by WGA staining. Graph (right) represents the relative size of cardiomyocytes in the UUO mice compared to controls. ***P < 0.001 vs. Control. Bar = 100 μm. Bar values represent means ± SEM (error bars). Statistical analyses were performed with t-tests. N = 8. (**D**) Representative immunofluorescence images of anti-CD-31 staining in the heart in control and UUO mice. Red fluorescence signal represents CD-31 and blue fluorescence signal represents DAPI. Graph represents quantification of cardiac vascular rarefaction. There was observed but not statistically significant cardiac vascular rarefaction in the UUO mice relative to the controls. *P* = 0.5980 vs. Control. Bar = 100 µm. Bar values represent means ± SEM (error bars). Statistical analyses were performed with t-tests. N = 8.

Cardiac hypertrophy was seen in UUO/CKD mice as indicated by an increased heart weight to body weight ratio (Fig. 3A,B). Cardiomyocyte cross-sectional analysis by immunofluorescence staining with WGA revealed that the average cardiomyocyte size was significantly increased in the UUO mice (Fig. 3C). Therefore, UUO injury caused an increase in cardiac mass and cardiomyocyte size. Vascular rarefaction was examined by anti-CD31 staining of UUO and control hearts. Through quantification of CD31-positive vessels we identified observable but not statistically significant vascular rarefaction in the UUO hearts (Fig. 3D).

**Figure 4:**
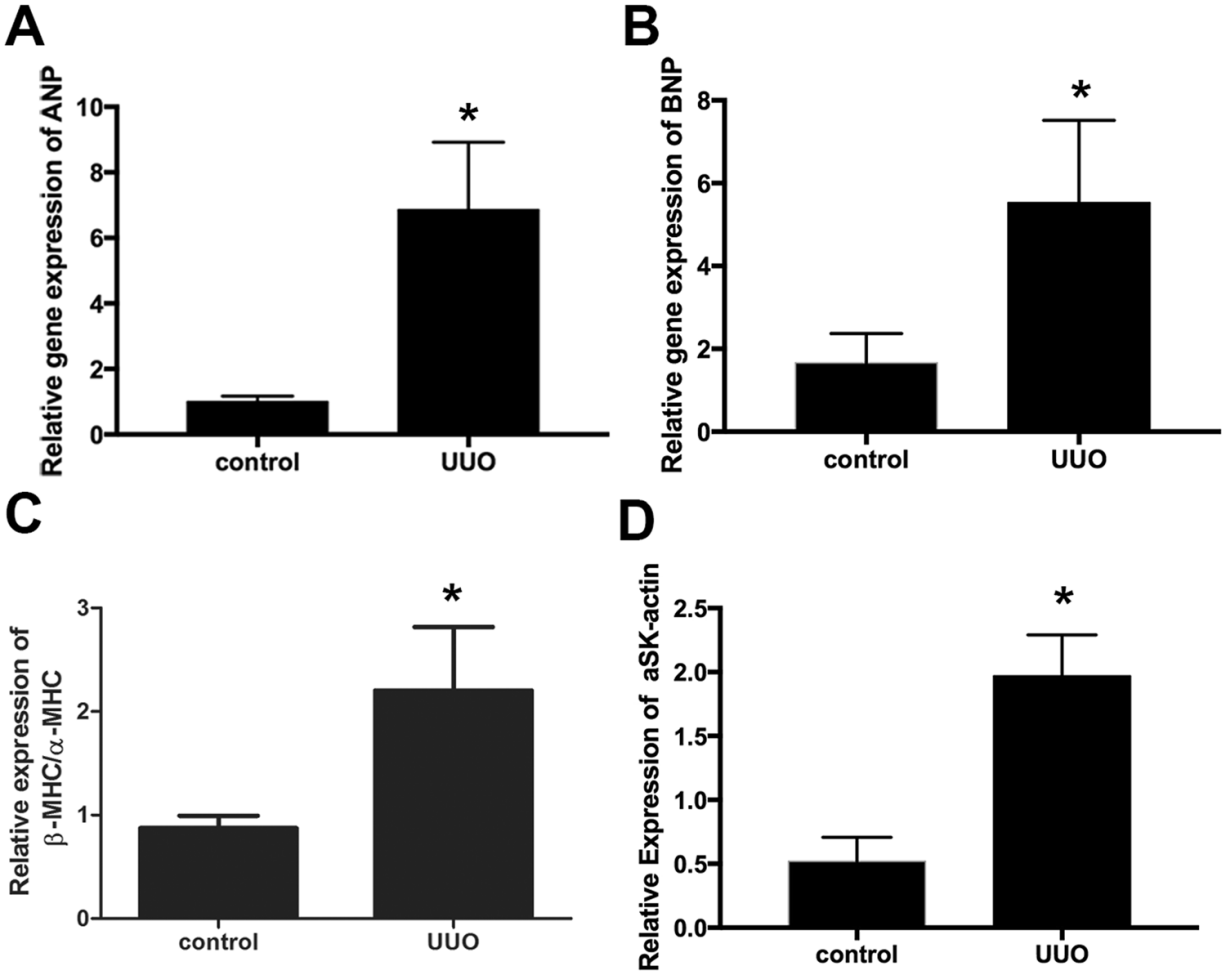
Expression of cardiac hypertrophy-related genes in UUO/CKD mice. The expressions of 4 major markers for cardiac hypertrophy, ANP, BNP, β-MHC, and α-SK-actin, were measured by qRT-PCR. There was significantly increased expression of ANP (**A**), BNP (**B**), β-MHC/α-MHC (**C**), and α-SK-actin (**D**) in mice with UUO-induced CKD compared to the controls. Expression levels of individual genes were normalized to the expression level of β-actin. *P < 0.05 vs. Control. Bar values represent means ± SEM (error bars). Statistical analyses were performed with t-tests. N = 7.

We further examined the expression of several key cardiac hypertrophy-related genes, using the quantitative real-time polymerase chain reaction (qRT-PCR, Fig. 4A–D). Our data showed that the expression of atrial natriuretic peptide (ANP), brain natriuretic peptide (BNP), α-skeletal (SK)-actin, and the ratio of β-myosin heavy chain (β-MHC)/α-MHC expression were all significantly increased in the UUO hearts, suggesting that possible pathological cardiac hypertrophy was induced by UUO injury in mice^37–39^.

### Cardiac fibrosis occurs in UUO-induced early CKD mice

**Figure 5:**
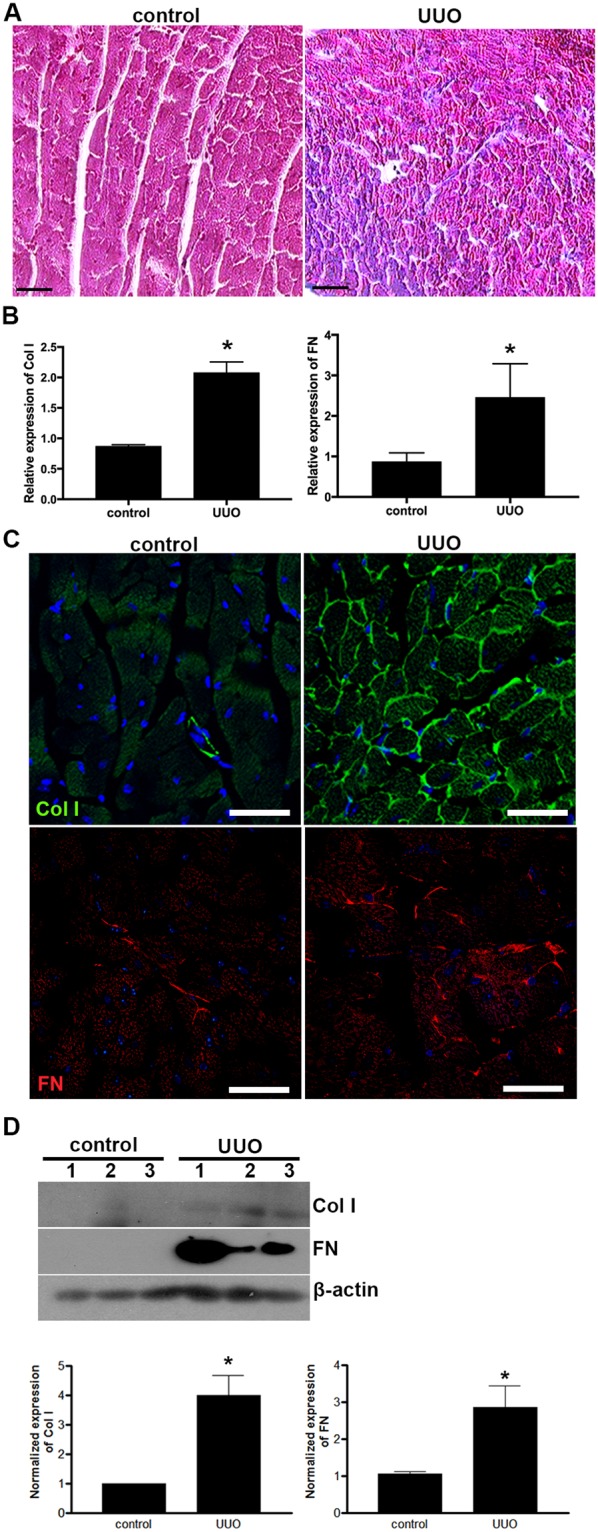
Cardiac fibrosis developed in UUO/CKD mice. (**A**) Masson’s trichrome staining of cardiac tissue revealed the presence of interstitial fibrosis in the UUO/CKD hearts. Increased fibrosis was substantial and diffuse in the UUO heart. Bar = 100 μm. (**B**) Expression of collagen type 1 and fibronectin was significantly elevated in the UUO/CKD hearts compared to the controls, as determined by qRT-PCR. The expression levels of individual genes were normalized to the expression level of β-actin. *P < 0.05 vs. Control. (**C**) Immunofluorescence staining for collagen type 1 (Col I) revealed diffusely increased collagen deposits in the UUO heart. In particular, there was a pattern of intercardiomyocytic accumulation of collagen type I in the UUO/CKD heart (upper panel). Immunofluorescence staining for fibronectin (FN) revealed increased accumulation of fibronectin in the interstitium and between cardiomyocytes in the UUO/CKD heart (lower panel). Bar = 100 μm. (**D**) Immunoblotting revealed significantly increased expression of collage type I and fibronectin in UUO/CKD hearts compared to the controls. The signal intensities of collagen type I and fibronectin were normalized to the signal intensity of β-actin, and presented in the graph (lower panel). *P < 0.05 vs. Control. Bar values represent means ± SEM (error bars). Statistical analyses were performed with t-tests. N = 9.

Pathological cardiac hypertrophy and cardiac fibrosis frequently co-exist^12–17^. Thus, we further examined whether UUO-induced cardiac hypertrophy is associated with any signs of cardiac fibrosis. We first performed Masson’s trichrome staining of cardiac tissues from control and UUO/CKD mice. Focal regions of fibrosis were frequently observed in cardiac tissue from UUO animals compared to the controls (Fig. 5A). We next looked at gene expression of several key ECM proteins including collagen type I and fibronectin. Our data showed that both collagen type I and fibronectin expression were nearly doubled in the hearts of UUO/CKD mice compared to the controls (Fig. 5B). Furthermore, immunofluorescence staining confirmed diffusely increased collagen type I staining around individual cardiomyocytes of the UUO/CKD mice. This intercardiomyocytic pattern of collagen type I staining is consistent with the previously reported pattern of “uremic intercardiomyocytic fibrosis” observed in uremic CKD patients^40^. In addition, we observed increased focal staining of fibronectin and collagen type I in the interstitium of the UUO hearts compared to the controls (Fig. 5C, upper and lower panel). This upregulation of collagen type I and fibronectin in cardiac tissue in UUO/CKD mice was further confirmed by immunoblotting, which showed approximately 4-fold and 1.5-fold increases in the expression of collagen type I and fibronectin, respectively, in the UUO heart (Fig. 5D). Collectively these data show that UUO injury induces cardiac hypertrophy and fibrosis in mice.

### UUO-induced cardiac fibrosis is associated with up-regulation of the TGF-β signaling pathway

**Figure 6:**
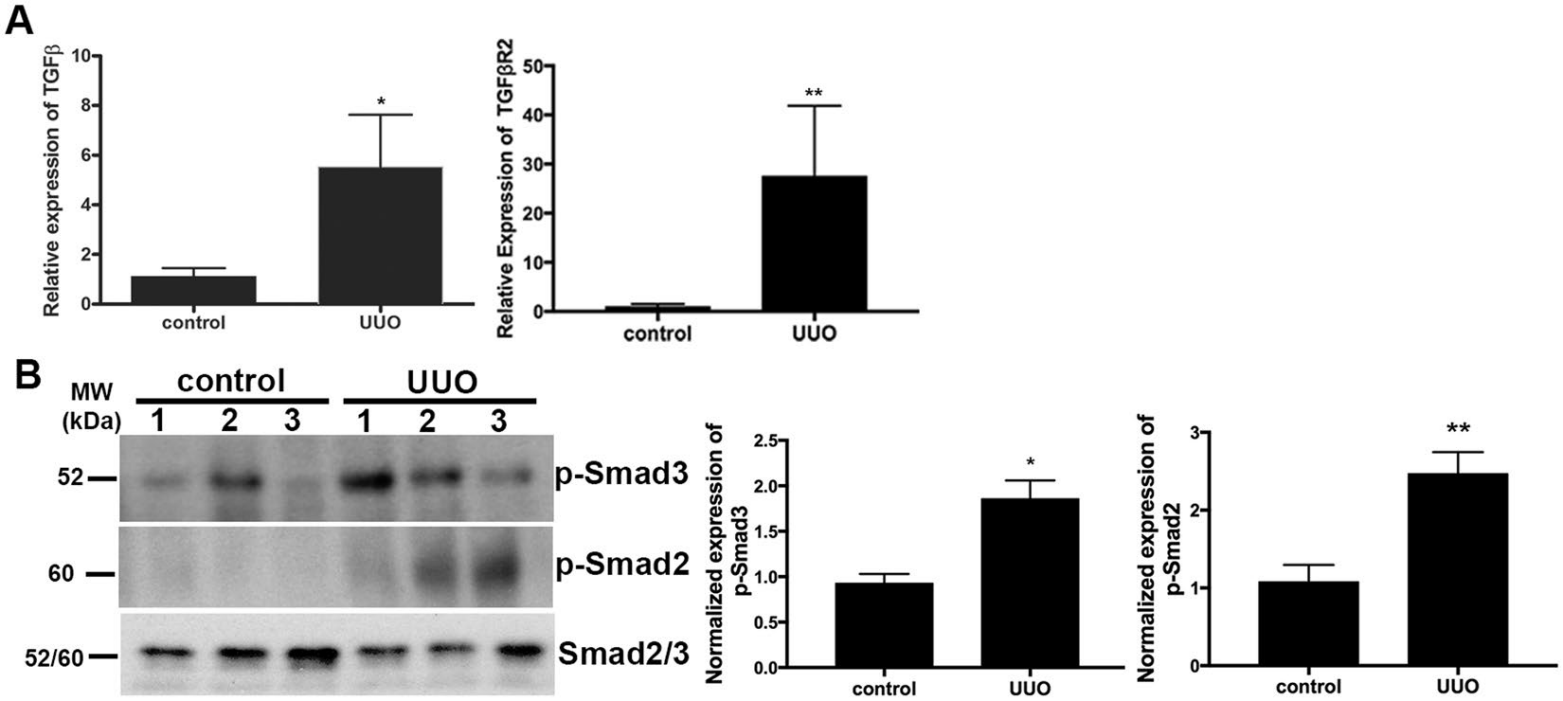
The TGF-β signaling pathway is activated in the hearts of UUO/CKD mice. (**A**) qRT-PCR revealed increased expression of TGF-β and TGF-β receptor 2 (TGF-β-R2) in the hearts of UUO/CKD mice. The expression levels of individual genes were normalized to the expression level of β-actin. *P < 0.05, **P < 0.01 vs. Control. (**B**) Immunoblotting revealed an increase in phosphorylated Smad2 and Smad3 in the hearts of UUO/CKD mice, indicating activation of the TGF-β signaling pathway. The expression levels of phosphorylated Smad2 and Smad3 were adjusted to the total level of Smad 2/3. Protein quantification was performed using ImageJ software. *P < 0.05, **P < 0.01 vs. Control. Values were expressed as means ± SEM (error bars). Statistical analyses were performed using t-tests. N = 6.

The TGF-β signaling cascade has been shown to be involved in many fibrotic processes, including cardiac fibrosis^12,41,42^. We next investigated the involvement of TGF-β signaling in UUO-induced cardiac fibrosis. We first examined the gene transcription of TGF-β and TGF-β-R2 using qRT-PCR (Fig. 6A). Our data showed that both TGF-β and TGF-β-R2 expression were significantly elevated in cardiac tissues of UUO/CKD mice compared to controls. Two key downstream effectors, Smad2 and Smad3, of the canonical TGF-β signaling pathway were further investigated by immunoblotting. The phosphorylation of Smad2 and Smad3 was increased almost 2-fold in the hearts of the UUO/CKD mice compared to the controls (Fig. 6B).

### Cardiac function and left ventricular mass are altered in mice with early stage CKD induced by UUO

Given the significant cardiac hypertrophy and fibrosis seen in non-uremic UUO/CKD mice, we performed echocardiography to evaluate cardiac structure and function in these mice. Our gravimetric assessment echocardiography showed an ~18% increase in LV mass in the UUO/CKD mice compared to controls (Table 1), which is consistent with the pathological cardiac hypertrophy seen in these animals. The cardiac function of mice was assessed by measuring the left ventricular ejection fraction (LVEF). Although not statistically significant, there was a trend toward reduced systolic function/LVEF in UUO/CKD mice (Table 1). It is possible that a sub-clinical cardiac dysfunction may be present in the UUO/CKD hearts, and is not yet clinically significant as measured using standard echocardiography.

**Table 1:** Echocardiography of control mice and mice with UUO injury.

Quantitative Parameters	Control	UUO	P-value
LVEDd (mm)	1.82 ± 0.11	2.10 ± 0.13	0.120
LVESd (mm)	0.91 ± 0.03	1.13 ± 0.05	0.001
LVAWd (mm)	1.091 ± 0.16	1.000 ± 0.04	0.190
LVPWd (mm)	1.446 ± 0.03	1.465 ± 0.04	0.710
LVM (mg)	74.06 ± 1.75	88.30 ± 2.71	0.001
LVEF (%)	85.18 ± 2.08	76.88 ± 6.54	0.079
HR (bpm)	622.14 ± 11.48	611.48 ± 48.48	0.647

LVEDd, left ventricular end diastolic diameter; LVESd, left ventricular end systolic diameter; LVAWd, left ventricular anterior wall dimension at end-diastole; LVPWd, left ventricular posterior wall thickness at end-diastole; LVM, left ventricular mass; LVEF, left ventricular ejection fraction; HR, heart rate.

### Inhibition of angiotensin II-converting enzyme attenuates pathological cardiac hypertrophy in UUO/CKD mice

The RAS plays an important role in mediating the pathological remodeling of multiple organ systems, including the heart and kidneys^43,44^. Inhibiting ACE has been shown to improve left ventricular hypertrophy (LVH) in patients and animals^22,45–47^. Thus we investigated the effect of Enalapril, an ACE I, in modulating pathological cardiac hypertrophy in UUO-induced CKD in mice.

**Figure 7:**
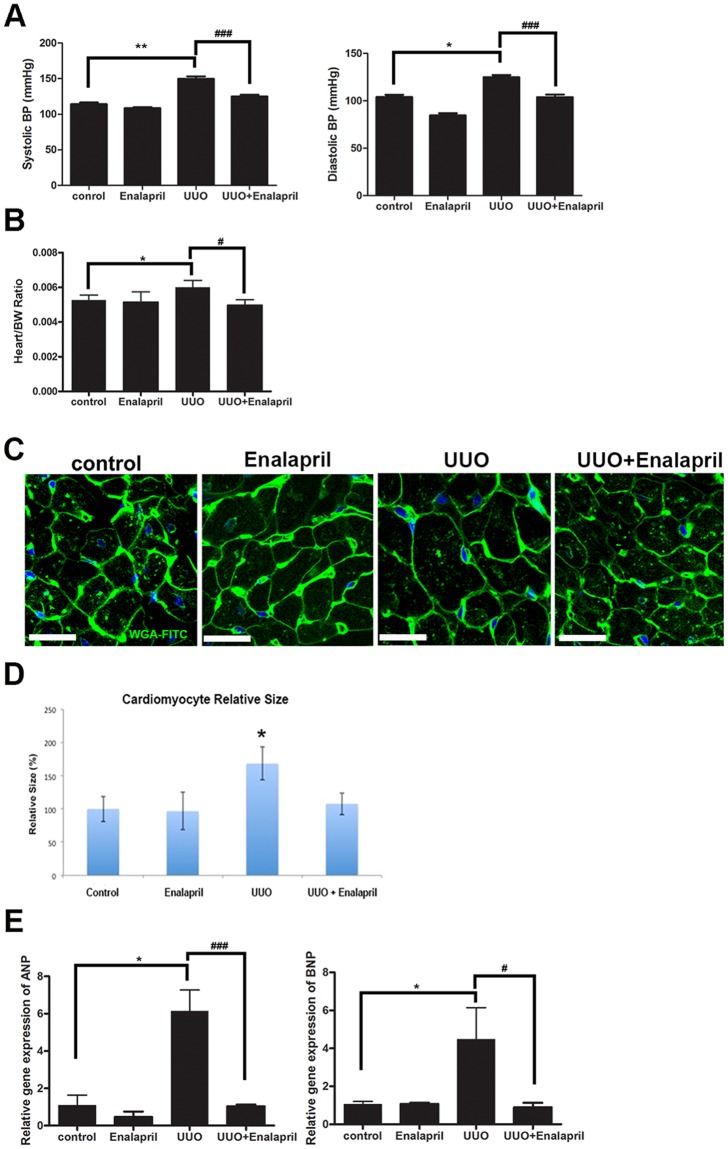
Enalapril decreases pathological cardiac hypertrophy in UUO/CKD mice. (**A**) Both systolic and diastolic BP were significantly elevated in the UUO-only mice compared to the Enalapril-treated UUO mice. The mean systolic BP was 144.92 mmHg and 125.96 mmHg in UUO/CKD and Enalapril-treated UUO/CKD mice, respectively. The mean diastolic BP was 124.78 mmHg and 106.27 mmHg in UUO/CKD and Enalapril-treated UUO/CKD mice, respectively. *P < 0.05 vs. Control, **P < 0.01 vs. Control, ^###^*P* < 0.001 vs. UUO. (**B**) The increase in cardiac mass as measured by the ratio of heart weight to body weight was significantly reduced in the Enalapril-treated UUO mice, relative to the UUO-only mice. *P < 0.05 vs. Control. ^#^*P* < 0.05 vs. UUO. (**C**) Immunofluorescence staining of cardiac tissues with WGA-conjugated FITC. Decreased cell size was observed in the hearts of Enalapril-treated UUO mice by WGA staining. Bar = 100 μm. (**D**) Quantification of cardiomyocyte size was performed using ImageJ. Enalapril treatment alleviated the cardiomyocyte enlargement in UUO hearts. *P < *0.05* vs. Control. (**E**) The expressions of cardiac hypertrophy genes ANP and BNP were measured by qRT-PCR. There was significantly decreased expression of ANP and BNP in the Enalapril-treated UUO mice. Expression levels of individual genes were normalized to the expression level of β-actin. *P < 0.05 vs. Control, ^#^*P* < 0.05 vs. UUO, ^###^*P* < 0.001 vs. UUO. Bar values represent means ± SEM (error bars). Statistical analyses were performed with t-tests. N = 6.

Mice were treated daily with Enalapril, starting one day after UUO for a total of 21 days. Treatment with Enalapril improved the blood pressures of UUO mice to the level of the control mice. Enalapril-treated UUO mice had an average 27.9 mmHg to 42.2 mmHg reduction in their systolic and diastolic BP’s respectively, compared to the UUO/CKD mice (Fig. 7A). The heart to body weight ratio was decreased in Enalapril-treated UUO/CKD mice compared to UUO-only/CKD mice, indicating reduced cardiac mass following Enalapril treatment (Fig. 7B). Cardiomyocyte cross-sectional analysis by immunofluorescence staining with WGA, followed by quantification with ImageJ, revealed a significant reduction in cardiomyocyte size following Enalapril treatment in UUO-CKD mice (Fig. 7C). We further examined the expression of genes associated with pathological cardiac hypertrophy using qRT-PCR. Enalapril treatment significantly reduced the up-regulation of ANP and BNP in UUO/CKD mice (Fig. 7D). We performed a similar experiment to examine the effect of hydralazine, a commonly used anti-hypertensive agent, on UUO-induced cardiac remodeling in comparison to Enalapril. Both hydralazine and Enalapril counteracted the UUO-induced increases in systolic and diastolic blood pressures. Unlike Enalapril, hydralazine did not improve serum creatinine, reduce cardiomyocyte size, or attenuate collagen deposition in the UUO heart (Supplemental Figure S1). Therefore our data indicates that Enalapril reduces the pathological cardiac hypertrophy induced by UUO/CKD in mice.

### Cardiac fibrosis and activation of TGF-β signaling is blocked by Enalapril in UUO/CKD mice

**Figure 8:**
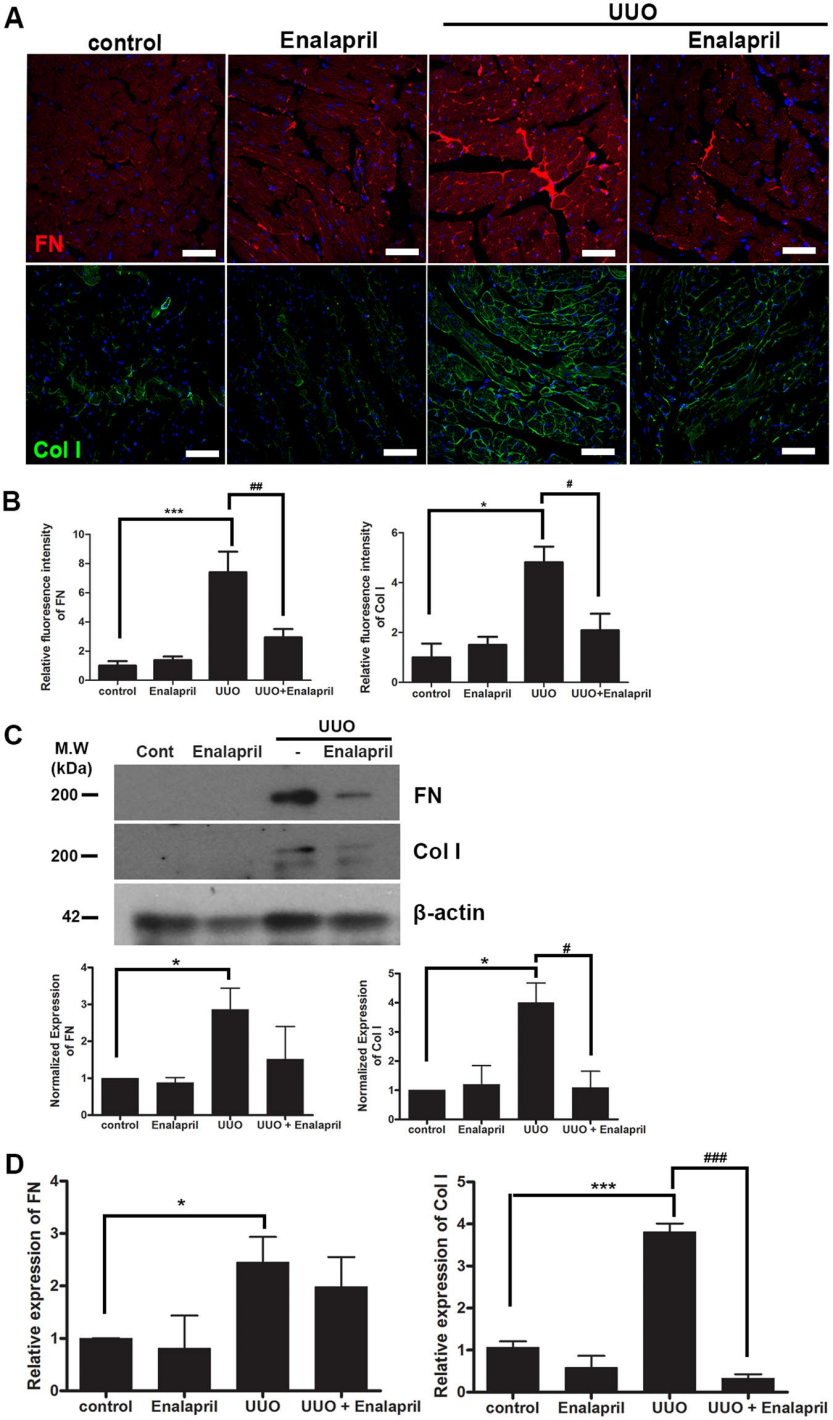
Enalapril attenuates cardiac fibrosis in UUO/CKD mice. (**A**) Immunofluorescence staining revealed that Enalapril treatment reduced intercardiomyocytic accumulation of collagen type I (Col I) and interstitial deposition of fibronectin (FN) in UUO/CKD mice. Bar = 100 μm. (**B**) Quantified relative fluorescence intensity showed decreased expression of Col I and FN in Enalapril-treated UUO mice. The relative fluorescence intensity was measured using ImageJ. *P < 0.05 vs. Control, ***P < 0.001 vs. Control, ^#^*P* < 0.05 vs. UUO, ^##^*P* < 0.01 vs. UUO. (**C**) Immunoblotting confirmed that Enalapril treatment inhibited the expression of FN and Col I in the hearts of UUO/CKD mice. The expression levels of fibronectin and collagen type I were normalized to the expression level of β-actin. Protein quantification was performed using ImageJ software. *P < 0.05 vs. Control, ^#^*P* < 0.05 vs. UUO. (**D**) Reduced expression of FN and Col I in Enalapril-treated UUO/CKD mice was determined by qRT-PCR. The expression levels of fibronectin and collagen type I were normalized to the expression level of β-actin. *P < 0.05 vs. Control, ***P < 0.001 vs. Control, ^###^*P* < 0.001 vs. UUO. Values were expressed as means ± SEM (error bars). Statistical analyses were performed with one-way ANOVA. N = 6.

We further examined the effect of Enalapril in modulating early cardiac fibrosis in non-uremic UUO/CKD mice. Early administration of Enalapril resulted in significant reduction in the expression of collagen type I and fibronectin in the hearts of Enalapril-treated UUO mice compared to UUO/CKD mice without Enalapril. (Figure 8A). Wild-type mice treated with Enalapril were also examined. Enalapril treatment did not alter the baseline expression of collagen type 1 or fibronectin, nor did it cause intercardiomyocytic or interstitial fibrosis in wild-type mice. Through quantification of immunofluorescence signals, our data revealed a 50% reduction in fibronectin signal and collagen type I staining in the hearts of Enalapril-treated UUO mice, compared to the untreated UUO/CKD mice (Fig. 8B). Furthermore there were approximately 0.5-times and 3-times reduction in protein levels of collagen type I and fibronectin, respectively, in the hearts of Enalapril-treated UUO mice (Fig. 8C). Expression of fibronectin or collagen type I was barely detectable in the hearts of control animals without UUO. We further examined gene expression of fibronectin and collagen type I using qRT-PCR. Our data showed that collagen type I expression was significantly decreased in Enalapril-treated UUO mice compared to UUO-only mice (Fig. 8D).

**Figure 9:**
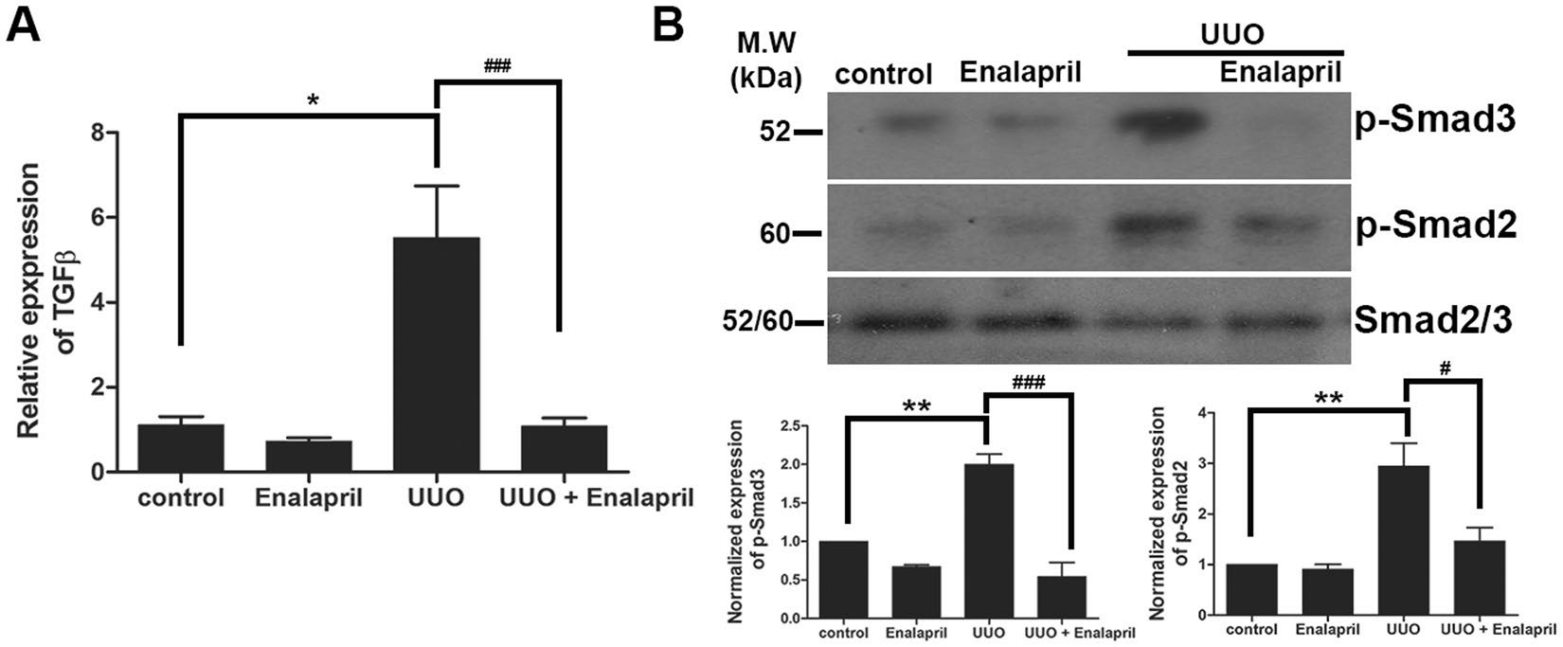
Enalapril treatment blocks activation of the TGF-β signaling pathway in UUO-CKD mice. (**A**) qRT-PCR revealed that Enalapril treatment reduced the expression of TGF-β mRNA in the hearts of UUO/CKD mice. The expression level of TGF-β was normalized to the expression level of β-actin. *P < 0.05 vs. Control, ^###^*P* < 0.001 vs. UUO. (**B**) Immunoblotting revealed that Enalapril treatment blocked UUO-induced phosphorylation of Smad2 and Smad3 in the hearts of UUO/CKD mice. Data were normalized to total Smad2/3. **P < 0.01 vs. Control, ^#^*P* < 0.05 vs. UUO, ^###^*P* < 0.005 vs. UUO. Values were expressed as means ± SEM (error bars). Statistical analyses were performed with one-way ANOVA. N = 6.

Angiotensin II has been reported to activate the TGF-β signaling pathway in various tissues and organs^48–51^. We have shown that UUO-induced early cardiac fibrosis is associated with the up-regulation of TGF-β signaling. We then examined whether the reduced cardiac fibrosis in Enalapril-treated UUO/CKD mice was associated with a decrease in TGF-β. Indeed the expression of TGF-β was significantly reduced in the hearts of Enalapril-treated UUO/CKD mice (Fig. 9A). The increased phosphorylation of Smad2 and Smad3, induced by UUO/CKD, was also significantly decreased in the hearts of Enalapril-treated UUO/CKD mice compared to untreated UUO/CKD mice (Fig. 9B). Therefore early ACE inhibition by Enalapril attenuates cardiac fibrosis and blocks the activation of the TGF-β signaling pathway in UUO/CKD mice.

## Discussion

Both the kidney and heart play vital roles in maintaining hemodynamics and homeostasis in the human body. The prevalence of both CKD and ESRD has been progressively increasing in developed countries, due to increasing rates of diabetes, obesity and hypertension^52,53^. It is widely reported that cardiac dysfunction is prevalent among CKD patients, especially those with ESRD who are undergoing dialysis^54–57^. Adverse cardiovascular events are currently the most common cause of death in CKD and ESRD patients. Effective management to reduce the high cardiac mortality and morbidity in the CKD and ESRD population is currently lacking, given limited understanding of the pathological mechanisms contributing to the development of cardiorenal syndrome. It was demonstrated in animals that severe CKD induced by radical nephrectomy (5/6 nephrectomy) caused diastolic dysfunction and ventricular hypertrophy at 4 weeks, and progressive cardiac fibrosis and expression of molecular signatures of heart failure after 8 weeks of kidney injury^58^. Myocardial dysfunction occurs prior to changes in ventricular geometry in mice with chronic kidney disease (CKD). However, many animal and clinical studies have focused on uremic cardiomyopathy in advanced CKD (type 4 and 5) or in patients on dialysis^28,29,59^.

Recognizing and understanding cardiac pathology and its underlying molecular mechanisms before the development of overt cardiac dysfunction is critical for delaying the initiation and progression of cardiovascular complications in advanced CKD patients. However, when and how pathological cardiac remodeling occurs in early stage CKD has not been well studied. Our study utilized the UUO injury model, which induces only mild-to-moderate CKD due to compensatory function from the non-injured kidney. Our data revealed that pathological cardiac hypertrophy and fibrosis occur early in mice three weeks after UUO, as evidenced by increased cardiomyocyte size and cardiac mass. We also found increased expression of pathological cardiac hypertrophy-related genes, including ANP, BNP, β-MHC/α-MHC, and α-SK-actin.

A maladaptive cardiac hypertrophic response develops frequently under pathological conditions such as uncontrolled hypertension, chronic volume overload and chronic anemia, conditions that are frequently associated with CKD. Indeed, LVH is the most common cardiac alteration observed in patients with CKD, and reaches 75% prevalence at the time of dialysis initiation^60–62^. This maladaptive LVH in advanced CKD and ESRD patients is commonly associated with cardiac fibrosis. Interestingly, multiple studies have revealed a unique form of cardiac fibrosis in patients with advanced CKD^18,63^. This form of “uremic” cardiac fibrosis presents as a pattern of uniform intermyocardial fibrosis, which differs from the endocardial to epicardial perivascular fibrosis seen in patients with hypertension and ischemic cardiomyopathies. This type of uremic “intercardiomyocytic fibrosis” is observed in over 90% of pre-dialysis CKD and ESRD patients, but absent in non-uremic controls in post-modem biopsy, therefore indicating a unique CKD-specific pathological process in CRS type 4^64^. Whether this uremic intercardiomyocytic fibrosis also occurs in non-uremic CKD is unknown. In our UUO-induced CKD mice, we have detected diffuse intermyocardial and interstitial fibrosis with significant deposition of fibronectin and collagen type 1. This is consistent with the reported uremic cardiac fibrosis. This diffuse intercardiomyocytic fibrosis is associated with activation of the canonical TGF-β/Smad 2/3 signaling pathway.

How does UUO/CKD induce cardiac hypertrophy and fibrosis? Cardiorenal syndrome is very complex and poorly understood^29,32,40^. As with other models of CKD-induced cardiomyopathy, the mechanism of CRS in the UUO-CKD model remains to be elucidated. In our model we have not detected any direct injuries such as apoptosis of cardiomyocytes in UUO/CKD hearts (data not shown). However, our data suggests the involvement of the RAS in the early pathological remodeling in the hearts of our UUO/CKD mice. Inhibiting the RAS with the ACE I Enalapril significantly blocks the activation of the TGF-β signaling cascade, and attenuates UUO/CKD-induced pathological cardiac hypertrophy and fibrosis. The RAS together with the autonomic nervous system (ANS), arginine vasopressin (AVP), and endothelin, constitute important regulatory mechanisms in the neurohormonal network and function to maintain hemodynamic stability as well as volume and electrolyte homeostasis^65–68^. In our study we did observe a significant reduction in blood pressure in UUO/CKD mice after treatment with Enalapril. Hypertension and volume overload may contribute to the development of uremic cardiomyopathy. However, more recent studies have demonstrated the role of “hypertrophic” and “fibrogenic” factors or signaling events in mediating uremic cardiomyopathy in CKD mice. For example, the involvement of the mammalian target of rapamycin (mTOR) pathway was reported in a pressure-controlled uremic mouse model^69^. Inhibiting mTOR activity with rapamycin reduced cardiac hypertrophy, but not through lowering of blood pressure^70^. Whether the observed protective effect of Enalapril against cardiac hypertrophy and fibrosis is through its anti-hypertensive effect or its direct effect on cardiac fibrogenesis in the UUO heart is not clear. We therefore performed a similar study using an antihypertensive reagent hydralazine on UUO/CKD mice. Interestingly, hydralazine did not significantly alleviate cardiac hypertrophy or fibrosis as Enalapril did in UUO/CKD mice. Therefore, it is possible that Enalapril may directly modulate fibrogenesis in the UUO/CKD heart.

For many years it has been well reported that RAS inhibition protects against renal interstitial fibrosis induced by UUO^69,71–73^. RAS activation has also been implicated in the development of cardiac fibrosis in CKD and ESRD patients^74–78^. Evidence from multiple randomized clinical trials supports the notion that inhibiting the RAS improves all causes of mortality and reduces adverse cardiovascular events and strokes, which is not commonly seen with other antihypertensive medications^64,79–81^. RAS inhibition has been widely utilized in managing patients with CVD, especially with co-morbidities of CKD or diabetes mellitus^82–87^. However, despite the fact that RAS inhibition provides systemic protection against tissue fibrosis in the heart and kidney, the use of ACE I is only suggested in patients with stable CKD or ESRD. Use of ACE I during the acute phase of kidney injury is not recommended. Our study has demonstrated a beneficial effect of ACE I in the early development of cardiac fibrosis in UUO/CKD mice. Early treatment with Enalapril is able to significantly reduce pathological cardiac hypertrophy and fibrosis, and block up-regulation of the canonical TGF-β signaling pathway. These findings are consistent with previously reported crosstalks between angiotensin II and the TGF-β signaling pathway^48,88^. More importantly, our animal data supports the potential benefit of ACE I in the management of early and pre-clinical CRS during early CKD.

Finally, given the relatively high prevalence of LVH, cardiac fibrosis, and high cardiovascular mortality in CKD patients, we emphasize that it is very important to recognize and detect the sub-clinical cardiac pathology in early CKD. Although the overall renal dysfunction may be variable and difficult to predict in the UUO model because of the compensatory function of the contralateral kidney, the UUO model enables us to detect the early impact of CKD on the development of cardiac remodeling before the overt clinical symptoms occur. Despite the clear presence of pathological cardiac hypertrophy and fibrosis in UUO/CKD hearts, we are not able to detect overt cardiac dysfunction through conventional approaches. Although we have not examined the cardiac reserve and peak performance in our UUO/CKD mice, a recent clinical study has demonstrated impaired peak cardiac performance and cardiac functional reserve in asymptomatic CKD patients in the absence of overt cardiac dysfunction. This indicates that an insidious pre-clinical cardiac pathological process occurs early in CKD patients^35^. Therefore, installing a “stress test” to monitor cardiac functional reserve in early stage CKD patients may be necessary to uncover any subclinical cardiac decompensation, which has been largely overlooked. Targeting an early pathological remodeling process will offer a more effective strategy to delay the initiation and progression of cardiac fibrosis and cardiomyopathy in CKD patients.

In summary, our study demonstrates the development of pathological cardiac remodeling and “uremic” cardiac fibrosis in mice with non-uremic CKD induced by UUO, in the absence of overt cardiac dysfunction. Early intervention with RAS inhibition is able to attenuate cardiac fibrosis and antagonize activation of the fibrogenic TGF-β signaling pathway in UUO/CKD mice, and highlights the importance of early recognition and intervention for pre-clinical CRS.

## Materials and Methods

### Reagents and chemicals

The reagents and chemicals were purchased from the following venders: BD Microtainer (ref. # 365956, BD Bioscience, Franklin Lakes, NJ), QuantiChrom^TM^ Creatinine Assay Kit (Cat#: DICT-500, Bioassay Systems, Hayward, CA), Tissue-Tek OCT Compound 4583 (Sakura Finetek USA, Torrance, CA), Bovine Serum Albumin (Santa Cruz Biotechnology, Dallas, TX), Trizol (Invitrogen, Buffalo, NY), Reverse Transcription System Kit (Biotool, Jupiter, FL). Powerup SYBR Green Master Mix (Applied Biosystems, Foster City, CA), and Buprenorphine Hydrochloride (Bedford Laboratories Bedford, OH). Formalin, Ethanol, and Xylene were purchased from Fisher Scientific Company L. L. C. (Kalamazoo, MI). Enalapril, Hematoxylin-Eosin (H&E) staining kit, and Masson’s trichrome staining kit were from Sigma-Aldrich (St. Louis, MO). Polyvinylidene difluoride (PVDF) membranes were from Millipore (Billerica, MA).

The commercial primary antibodies used were from the following companies: anti-collagen type I (Sigma-Aldrich), anti-fibronectin (Sigma-Aldrich), anti-CD31 (Santa Cruz Biotechnology, Dallas, TX), anti-β-actin (Sigma-Aldrich), anti-phospho-Smad-2 (Cell Signaling Technology, Danvers, MA), anti-phospho-Smad-3 (Cell Signaling Technology), anti-Smad-2/3 (Santa Cruz Biotechnology), and anti-wheat germ agglutinin (WGA; Vector Laboratory, Burlingame, CA). Secondary conjugated indocarbocyanine (Cy3) and fluorescein isothiocyanate (FITC) antibodies were obtained from Jackson Immunoresearch Laboratories (West Grove, PA). Commercial secondary HRP-conjugated donkey anti-rabbit and donkey anti-mouse antibodies were purchased from Santa Cruz Biotechnology.

### Animal experiments

Pathogen-free, 10–12 week old male C57BL/6 mice of approximately 25 g body weight were purchased from Jackson Laboratory (Bar Harbor, ME). All animal experiments were conducted according to the National Institutes of Health (NIH) Guide for the Care and Use of Laboratory Animals, and were approved by the Massachusetts General Hospital (MGH) Subcommittee on Research Animal Care. They were housed in temperature-controlled conditions, with proper humidity, lighting (12 hours light/12 hours dark cycle), and free access to food and water. The study was performed according to the guidelines developed by the Institutional Animal Care and Use Committee of MGH. All efforts were made to minimize animal suffering.

The mice were randomly assigned into six groups. There were 12 animals each in the control, unilateral urinary obstruction (UUO) only, and UUO treated with Enalapril groups. There were 7 animals each in the hydralazine and UUO treated with hydralazine groups. 10 animals were also used in another group that received Enalapril only. All procedures were performed under anesthesia with 3% isoflurane inhalation. 0.1 mg/kg of buprenorphine hydrochloride was injected subcutaneously at the surgical site before, and every 8 to 12 hours after, the surgical procedure to ease any local pain or discomfort. For the UUO operation the left kidney was exposed through a left flank incision, then the left ureter was ligated with 5-0 silk at two sites between the bladder and renal pelvis. The mice were observed closely after surgery. Food and water intake and body weight were also monitored. The dosage of Enalapril was selected based on reported animal studies^89,90^ 15 mg/kg Enalapril and 5 mg/kg hydralazine^91^ were administered daily by intraperitoneal injection for three weeks, starting on the second day after UUO injury. Mice were monitored closely for 3 weeks after left ureter ligation. Echocardiograms were performed 3 weeks after UUO prior to harvesting tissues. Serum creatinine was also measured. Kidneys and hearts were collected, weighed, and processed for histological examination and protein and gene analysis. All the mice survived through the procedures. Mice were harvested three weeks after the UUO procedure. 0.5–0.7 ml of blood was collected from the inferior vena cava using an insulin syringe. Heart and kidney tissues were immersed and fixed in PLP containing 4% paraformaldehyde, 10 mM lysine, 10 mM periodate, 5% sucrose, and 0.1 mM sodium phosphate overnight in preparation for cryosection. Tissues were also fixed in 10% formalin for paraffin embedding. After fixation overnight at 4 °C all tissues were washed with PBS (10 mM sodium phosphate buffer containing 0.9% NaCl, pH 7.4) every 2 hours for 8 hours at 4 °C. All tissues were stored in PBS containing 0.02% NaN_3_ at 4 °C until use. Some heart and kidney tissues were also snap frozen in liquid nitrogen and stored at −80 °C for use in protein and RNA extraction.

### Immunofluorescence staining

After fixation mouse hearts were incubated in 30% sucrose/PBS overnight, then embedded in OCT compound 4583. Cryosectioning (5 μm) of heart tissue was conducted using the CM3050S cryostat (Leica Microsystems, Bannokburn, IL), collected onto Superfrost Plus microscope slides (Fisher Scientific, Pittsburgh, PA), and stored at −20 °C until use.

Immunofluorescence staining was performed as previously described^92–94^. Tissue sections were rehydrated in PBS for 5 minutes, then treated with 1% SDS/PBS for 4 minutes. After washing with PBS tissue slides were blocked with 1% BSA/PBS for 20 minutes. After blocking slides were incubated with primary antibodies at 4 °C for overnight. The antibodies that were used for this study were anti-collagen type I (1:2000), anti-fibronectin (1:2000), anti-CD31 (1:500), and anti-WGA (1:2000). All antibodies were diluted in 1% BSA/PBS. FITC or Cy3-conjugated donkey anti-mouse and anti-rabbit IgG (1:800) were used as secondary antibodies. The slides were mounted with DAPI-containing mounting medium (Vector Laboratory). All images were obtained using Zeiss LSM 800 (Carl Zeiss Microscopy GmbH, Jena, Germany) or A1R confocal laser-scanning microscopy (Nikon, Tokyo, Japan). Imaging analysis was performed using the software package ZEN 2.3 (Carl Zeiss) and NES (Nikon), and analyzed by NEI Element 3.0 (Nikon). Image brightness and contrast were linearly adjusted and a high pass filter was applied for removing noise, using Photoshop software (Adobe Systems Inc., San Jose, CA). Fluorescence intensity was also determined by ImageJ software (NIH, Bethesda, MD), normalized by control data, and analyzed statistically. Cardiomyocyte cell size was also determined using ImageJ.

### Hematoxylin-Eosin (H&E) and Masson’s trichrome staining

Hematoxylin and Eosin (H&E) and Masson’s trichrome staining were carried out as previously described^93,95^. After fixation with 10% formalin and washing with PBS, kidneys and hearts were dehydrated and prepared for paraffin embedding through an automated Leica TP1020 (Leica Microsystems, Wetzlar, Germany), then embedded in paraffin blocks at 60 °C. Tissue sections of 5 μm thickness were obtained using Jung RM2025 (Leica Microsystems). Tissue slides were deparaffinized with xylene, rehydrated in graded ethanol (100%, 70%, and 30%), and washed with PBS. Tissues slides were stained using the H&E staining kit according to the manufacturer’s instructions. Slides were incubated in hematoxylin for 5 minutes, then washed and stained with eosin for 5 minutes. After dehydration in ethanol and xylene, slides were mounted in Cytoseal 60 (Thermos Scientific). Tissue morphology was examined using Eclipse Ci microscopy (Nikon).

Cardiac fibrosis was examined by Masson’s trichrome staining using the commercial Masson’s trichrome staining kit and following the manufacturer’s instructions (Sigma-Aldrich). After de-paraffinization, tissue slides were fixed in Bouin’s solution and incubated in Weigert’s iron hematoxylin solution. Slides were then stained with Biebrich Scarlet-Acid Fuchsin and Aniline Blue. After dehydration in ethanol and xylene, slides were mounted and viewed. Collagenous material stained blue, cytoplasm and muscle fibers stained red, and nuclei stained black. Slides were examined and imaged using Eclipse Ci microscopy (Nikon).

### Vascular rarefaction

Vascular rarefaction was calculated as previously described^96^. Briefly, immunofluorescence images were overlaid onto 36 × 28 unit grids using Adobe Photoshop (Adobe Systems). For each image the number of squares with CD-31 signal present was determined by the naked eye. CD31-positive counts were averaged across 10 images each from the control and UUO treatments. Data entry, graph construction, and statistical analysis were performed using Microsoft Excel (Microsoft, Redmond, WA) and the Prism software (GraphPad Software, La Jolla, CA). Statistical analysis was performed using Student’s t-test and a *P*-Value of 0.05 or lower was considered significant.

### Immunoblotting

Immunoblotting was performed as previously described^93^. A total of 20 μg of tissue lysate was separated on 10% SDS-PAGE and transferred to PVDF membranes. Membranes were blocked with 5% non-fat milk for one hour and incubated overnight at 4 °C with primary antibody. Antibodies used for this study were anti-β-actin (1:10,000), anti-phospho-Smad-2 (1:1000), anti-phospho-Smad-3 (1:1000), anti-Smad-2/3 (1:2000), anti-collagen type I (1:2000), and anti-fibronectin (1:5000), followed by HRP-conjugated secondary antibody (1:10,000). To accommodate the low affinity of the anti-phospho-Smad 2 and 3 antibodies, we used large amounts of lysate for immunoblotting. After probing with anti-phospho-Smad antibodies, the membranes were stripped with stripping buffer (25 mM glycine-HCl, pH 2) for 40 minutes, blocked again with 5% non-fat milk for 1 hour, and probed with antibodies of higher affinity, including anti-β-actin (1:10,000), anti-collagen type I (1:2000), and anti-fibronectin (1:5000). The immunoblots were visualized using enhanced chemiluminescence (ECL) reagents. The intensity of each protein band was quantified using ImageJ software and analyzed statistically. The experiment was repeated at least three times.

### Quantitative real-time polymerase chain reaction (qRT-PCR)

Total RNA was extracted from mouse hearts using Trizol. Complementary DNA (cDNA) synthesis was carried out using the Reverse Transcription System Kit according to the manufacturer’s instructions (Biotool). One microgram of RNA was reverse-transcribed in a 20 μl reaction solution for 10 minutes at 25 °C, 30 minutes at 42 °C, and 5 minutes at 85 °C. PCR primers were synthesized by the MGH CCIB DNA Core Facility (Boston, MA). The primers that were used for qRT-PCR are summarized in Table 2. After obtaining cDNA, qRT-PCR was performed using the QuantStudio 3 machine and Powerup SYBR Green Master Mix (Applied Biosystems). The qRT-PCR reaction was set to start at 95 °C for 3 minutes, go through 40 cycles of denaturation, annealing, and extension, and end at 72 °C for 10 minutes as the final extension. Each qRT-PCR cycle involved 95 °C for 30 seconds, 55 °C for 30 seconds, and then 72 °C for 90 seconds. The threshold cycle (Ct) of each target gene, which was located in the linear amplification phase of the PCR, was measured automatically and normalized to the cycle number of the β-actin control. The relative expression levels of each mRNA were calculated (^ΔΔ^Ct) and reported as a fold induction (2^−ΔΔCt^).

**Table 2:** Primer list for real-time PCR.

Primer		Primer (5′-3′)
ANP	**F**	GCTTCCAGGCCATATTGGAG
	**R**	GGGGGCATGACCTCATCTT
BNP	**F**	GAGGTCACTCCTATCCTCTGG
	**R**	GCCATTTCCTCCGACTTTTCTC
α-MHC	**F**	GCCCAGTACCTCCGAAAGTC
	**R**	GCCTTAACATACTCCTCCTTGTC
β-MHC	**F**	ACTGTCAACACTAAGAGGGTCA
	**R**	TTGGATGATTTGATCTTCCAGGG
α-SK-actin	**F**	CCCAAAGCTAACCGGGAGAAG
	**R**	CCAGAATCCAACACGATGCC
Collagen type I	**F**	GCTCCTCTTAGGGGCCACT
	**R**	CCACGTCTCACCATTGGGG
Fibronectin	**F**	CTGGAGTCAAGCCAGACACA
	**R**	CGAGGTGACAGAGACCACAA
TGF	**F**	CCTCACCTCCATGTACCAGAA
	**R**	TGGAAATGACCTTGTCAATGAG
TGF-β-R2	**F**	CCGCTGCATATCGTCCTGTG
	**R**	AGTGGATGGATGGTCCTATTACA
β-actin	**F**	GTTGGCATAGAGGTCTTTAG
	**R**	GCCCGCATCCTCTTCCTCCCT

### Measurement of serum creatinine and blood urea nitrogen (BUN)

Serum creatinine was measured using the QuantiChrom^TM^ Creatinine Assay Kit according to the manufacturer’s instructions^91^. Serial dilutions of the standard, from 0.1 to 2 mg/dL, were prepared in a 96-well clear-bottom plate in order to generate the standard curve. 30 μl of serum was added to each well in triplicate. Then 200 μl of working solution was added to each well and mixed. The optical density was measured in SpectraMaxx (Molecular Devices, Sunnyvale, CA) at 490–530 nm. The mean peak absorbance was measured at 510 nm (OD sample/standard 5). Creatinine concentration of the sample was calculated using the following formula: [(OD sample 5 − OD sample 0)/(OD standard 5 − OD standard 0)] × [STD] (mg/dL).

Blood urea nitrogen (BUN) was measured using the Stanbio Urea Nitrogen Kit No. 0580 (Stanbio Laboratory, Boerne, TX). Briefly, serum samples and standards were mixed with BUN reagents in the appropriate proportion as specified by the manufacturer. This mixture was incubated in a 100 °C heat block for 12 min and then cooled to 0 °C for 5 min. The absorbance was read at 520 nm. The BUN levels of the samples were quantified using the standard curve generated by the standards.

### Blood pressure measurements

Blood pressure was measured in conscious mice using a non-invasive volume-pressure recording technique (CODA, Kent Scientific Corporation, Torrington, CT) as previously described^92^. Mice were allowed to acclimate to the system through training one day before the measurements. After several training sessions animals became comfortable with the environment and equipment, with no signs of agitation or stress. Measurements were performed after this acclimation period. Blood pressure was measured in a quiet and warm environment. It was performed at the same time every day by the same researcher, who trained the animals to minimize any variation. A minimum of five measurements were obtained for each mouse at each time point.

### Echocardiography analysis

Transthoracic echocardiograms were performed in conscious mice as previously described^27^. Briefly, echocardiographic images were acquired using a GE Vivid E90 system equipped with a high frequency L8-18i-D probe (14.0 MHz, GE Healthcare, Chicago, IL). M-mode images of the parasternal short axis were acquired at the papillary muscle level. Data was then analyzed using GE EchoPACS software. LV mass was calculated using the following formula: LV mass = [(LVAWd + LVEDd + LVPWd)^3^ − LVEDd^3^) × 1.05, where 1.05 is the specific gravity of heart muscle. Left ventricular function was presented as the ejection fraction (EF) calculated by the following formula: EF = [(LVEDd^3^ − LVESd^3^)/LVEDd^3^] × 100.

### Statistical analysis

Statistical analyses were performed using GraphPad Prism Version 5.0 (GraphPad Software, La Jolla, CA). Values were expressed as means ± SEM. Comparisons between two groups were performed with the two-tailed unpaired t-test. Multiple comparisons were analyzed using ANOVA followed by Bonferroni-corrected *post hoc* test. A significance level of *P* < 0.05 was defined as statistically significant.

## Electronic supplementary material

Supplementary Information
