# Supplementary material for: Pathological cardiac remodeling occurs early in CKD mice from unilateral urinary obstruction, and is attenuated by Enalapril
Source: Sci Rep. 2018 Oct 31;8:16087. doi: 10.1038/s41598-018-34216-x (PMC6208335; doi:10.1038/s41598-018-34216-x)
Supplement: Supplementary file 1 — Supplementary Information [file 41598_2018_34216_MOESM1_ESM.pdf]

Pathological cardiac remodeling occurs early in CKD mice from unilateral urinary obstruction, and is attenuated by Enalapril

Onju Ham[1#], William Jin[3#], Lei Lei[1,2], Hui Hui Huang[1], Kenji Tsuji[1], Ming Huang[1,2], Jason Roh[4], Anthony Rosenzweig[4], Hua A. Jenny Lu[1]

[1] Center for Systems Biology, Program in Membrane Biology, Division of Nephrology, Massachusetts General Hospital and Harvard Medical School, Boston, MA, USA, 02114.

[2] Department of Pharmacology, School of Basic Medical Sciences, Peking University, Beijing, China.

[3] College of Arts & Sciences, Washington University in St. Louis, St. Louis, MO, USA, 63130.

[4] Corrigan Minehan Heart Center, Massachusetts General Hospital and Harvard Medical School, Boston, MA, USA, 02114.

# Authors contributed equally.

Correspondence to Hua A. Jenny Lu, M.D., Ph.D., Division of Nephrology, Massachusetts General Hospital, Boston, MA 02114. E-mail: halu@partners.org

Keywords: UUO, CKD, Cardiac Hypertrophy, Cardiac Fibrosis, TGF-β Signal, Angiotensin-Converting-Enzyme Inhibitor

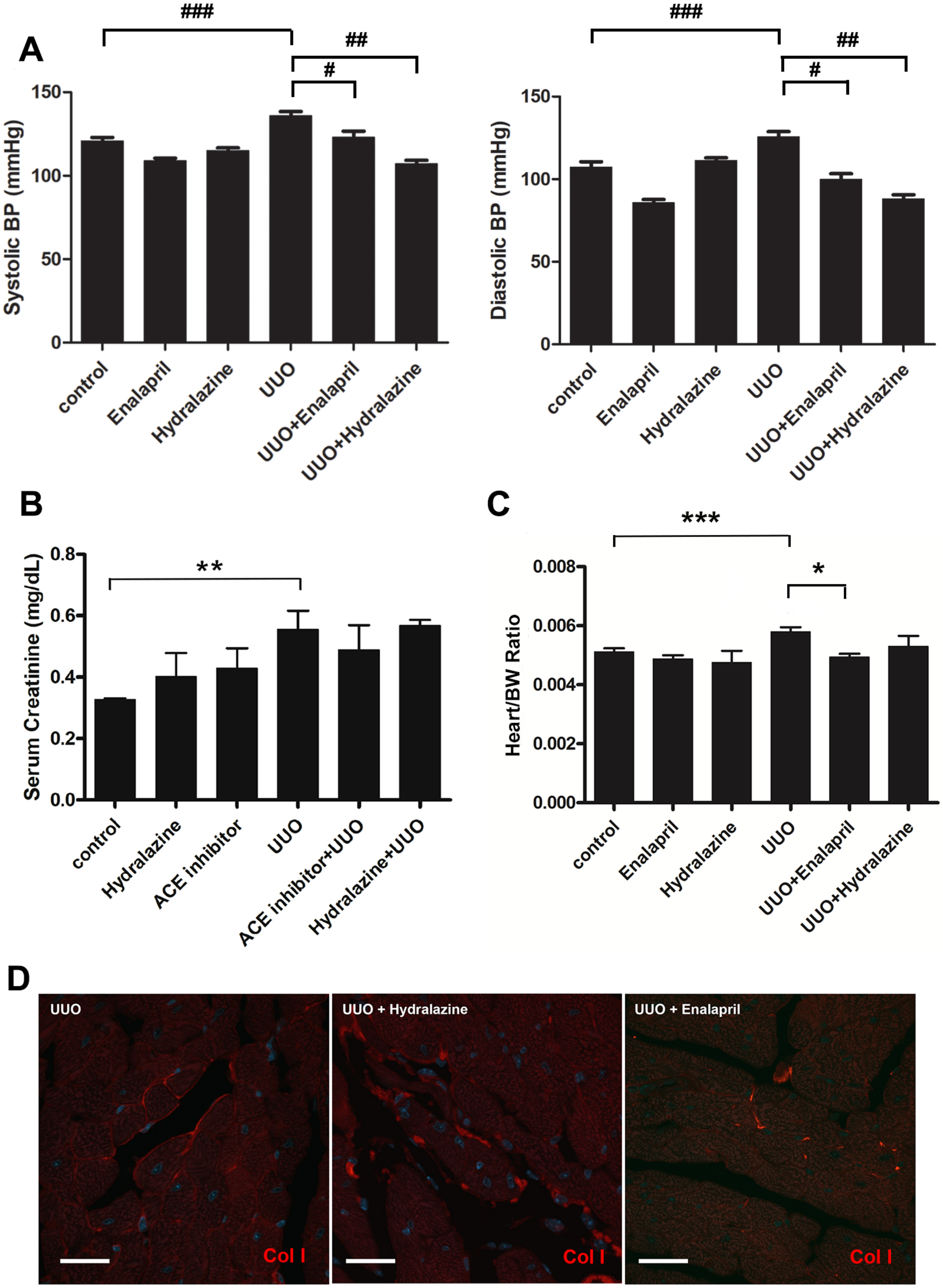

A
###
##
#
Systolic BP (mmHg)
150
100
50
0
control
Enalapril
Hydralazine
UUO
UUO+Enalapril
UUO+Hydralazine
###
##
#
Diastolic BP (mmHg)
150
100
50
0
control
Enalapril
Hydralazine
UUO
UUO+Enalapril
UUO+Hydralazine
B
**
Serum Creatinine (mg/dL)
0.8
0.6
0.4
0.2
0.0
control
Hydralazine
ACE inhibitor
UUO
ACE inhibitor+UUO
Hydralazine+UUO
C
***
*
Heart/BW Ratio
0.008
0.006
0.004
0.002
0.000
control
Enalapril
Hydralazine
UUO
UUO+Enalapril
UUO+Hydralazine
D
UUO
UUO + Hydralazine
UUO + Enalapril
Col I
Col I
Col I

Supplemental Figure S1. Effect of hydralazine treatment on cardiac hypertrophy and fibrosis in UUO mice. (A) Systolic and diastolic blood pressures were significantly elevated in the UUO mice compared to controls. Both Enalapril and hydralazine treatment corrected the blood pressure elevations in UUO mice. #*P* < 0.05, ##*P* < 0.01, ###*P* < 0.05, N = 7. (B) Serum creatinine was significantly elevated after UUO injury in mice. Enalapril treatment improved serum creatinine in UUO mice, while hydralazine treatment did not. ***P* < 0.01. N = 7. (C) Enalapril treatment significantly reduced heart-to-body-weight ratios in UUO mice, while hydralazine treatment did not. **P* < 0.01 vs. UUO, ****P* < 0.05 vs. Control. (D) Immunofluorescence staining for collagen type 1 (Col I) revealed that Enalapril treatment substantially reduced Col I deposition in the UUO heart, while hydralazine treatment did not. Red fluorescence represents collagen type I. Scale Bar = 100 µm. Values were expressed as means ± SEM (error bars). Statistical analyses were performed with one-way ANOVA. N=7.
